# Surveillance of avian influenza viruses from 2009 to 2013 in South Korea

**DOI:** 10.1038/s41598-021-03353-1

**Published:** 2021-12-14

**Authors:** Jeong-Hyun Nam, Erica Españo, Eun-Jung Song, Sang-Mu Shim, Woonsung Na, Seo-Hee Jeong, Jiyeon Kim, Jaebong Jang, Daesub Song, Jeong-Ki Kim

**Affiliations:** 1 Department of Pharmacy, Korea University College of Pharmacy, Sejong, 30019 Republic of Korea; 2 Division of Acute Viral Diseases, Center for Emerging Virus Research, National Institute of Health, Korea Disease Control and Prevention Agency, Cheongju, Chungbuk 28159 Republic of Korea; 3 Laboratory Animal Medicine, College of Veterinary Medicine, Chonnam National University, Gwangju, 61186 Republic of Korea

**Keywords:** Virology, Influenza virus, Viral reservoirs

## Abstract

Avian influenza viruses (AIVs) are carried by wild migratory waterfowl across migratory flyways. To determine the strains of circulating AIVs that may pose a risk to poultry and humans, regular surveillance studies must be performed. Here, we report the surveillance of circulating AIVs in South Korea during the winter seasons of 2009–2013. A total of 126 AIVs were isolated from 7942 fecal samples from wild migratory birds, with a total isolation rate of 1.59%. H1‒H7 and H9‒H11 hemagglutinin (HA) subtypes, and N1‒N3, N5, and N7‒N9 neuraminidase (NA) subtypes were successfully isolated, with H6 and N2 as the most predominant HA and NA subtypes, respectively. Sequence identity search showed that the HA and NA genes of the isolates were highly similar to those of low-pathogenicity influenza strains from the East Asian-Australasian flyway. No match was found for the HA genes of high-pathogenicity influenza strains. Thus, the AIV strains circulating in wild migratory birds from 2009 to 2013 in South Korea likely had low pathogenicity. Continuous surveillance studies such as this one must be performed to identify potential precursors of influenza viruses that may threaten animal and human health.

## Introduction

Avian influenza viruses (AIVs) are perpetuated in populations of wild aquatic birds, which are thought to be the primary reservoir or natural hosts of AIVs^1,2^. In wild bird species, AIVs are divided into 16 hemagglutinin (HA) and 9 neuraminidase (NA) subtypes based on serogrouping, and generally cause asymptomatic or mild disease^3^. Although wild aquatic birds typically carry low pathogenic avian influenza (LPAI), transmission and adaptation of these viruses to other bird species (e.g., domestic fowl) and to non-avian species (i.e., mammals) may result in respiratory disease^4^. Transmission of AIVs from wild waterfowl to poultry may arise from direct contact between the animals or their contaminated environments^5^. Poultry in farms infected with highly pathogenic avian influenza (HPAI) viruses are typically culled to prevent further spread of the virus, resulting in large agricultural and economic losses to affected locations^6^.

Among the HA subtypes that have the ability to cross the mammalian species barrier, two HA subtypes, H5 and H7, are associated with high pathogenicity^7^. Notably, HPAI H5N1 and H7N9 viruses are genetic reassortants of LPAI segments^8,9^. Thus, LPAI viruses are potential precursors of novel HPAI viruses. However, since it is virtually impossible to prevent the transmission of influenza A virus from wildfowl to domestic and wild animals, continuous surveillance of wild birds must be conducted to monitor and detect strains that have the potential to cause disease in both animals and humans.

In this study, we report the surveillance of AIVs in wild birds in South Korea during the winter seasons of 2009 to 2013. We isolated fecal samples from migratory bird stopover sites in South Korea, which is part of the East Asian-Australasian flyway (EAAF), and performed nucleotide sequence analysis to determine the distribution of AIVs among migratory wildfowl in South Korea.

## Results

### Prevalence of avian influenza viruses

**Figure 1:**
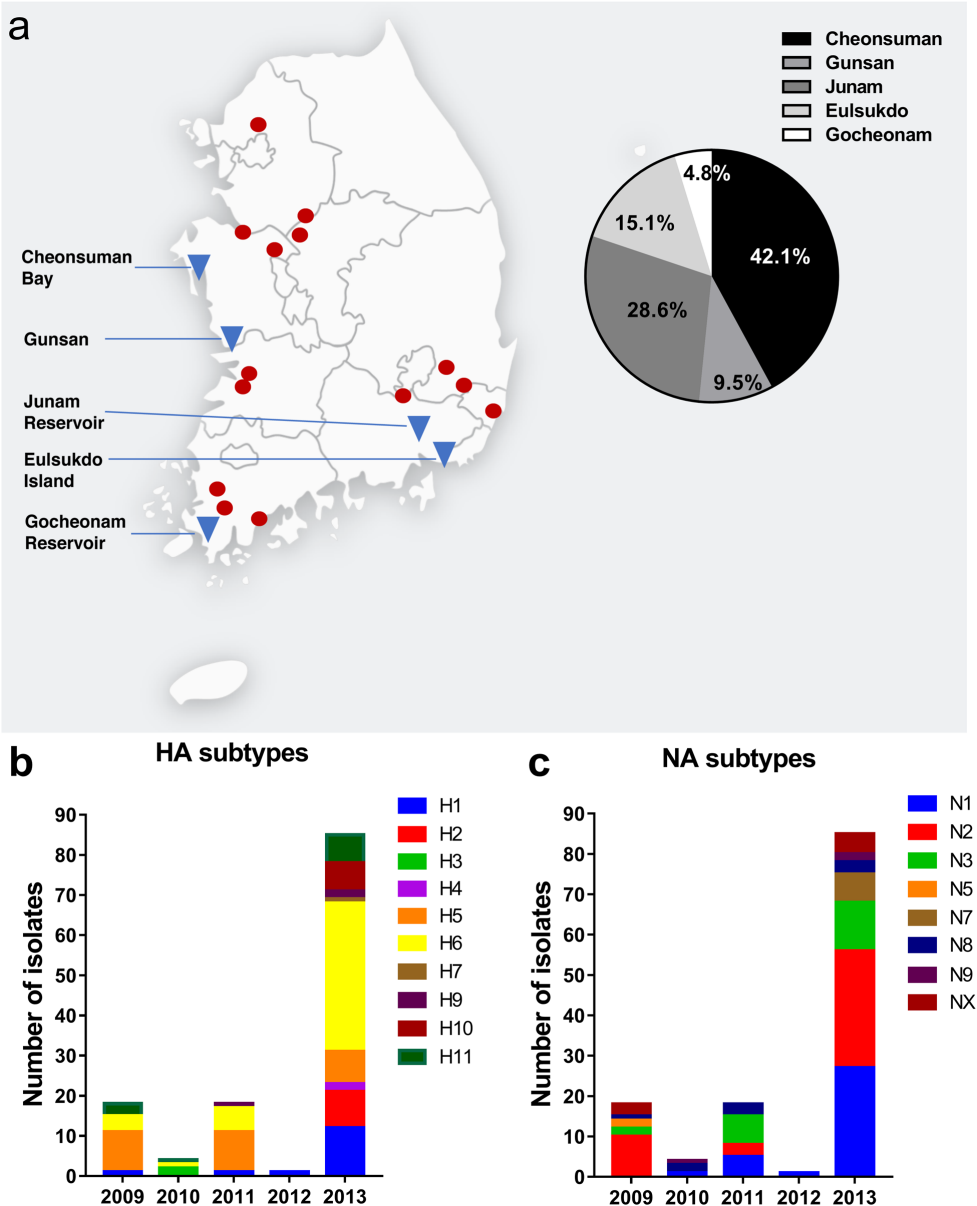
Sampling sites and hemagglutinin (HA) and neuraminidase (NA) subtypes of isolated avian influenza viruses from the fecal matter of wild migratory birds from 2009–2013. (**a**) Sampling sites (triangles) from 2009–2013, and representative clusters of highly pathogenic avian influenza outbreaks in poultry in South Korea from 2008–2014 (circles). Frequencies of isolates (out of 126 total isolates) per sampling site are shown in the pie chart. All (**b**) HA and (**c**) NA subtypes were identified through reverse transcription polymerase chain reaction using universal primers.

During the winter seasons of 2009‒2013, a total of 7942 wild bird fecal samples were collected from wild migratory bird habitats in South Korea (Fig. 1a). HPAI isolates have been obtained from wildfowl in these sites, and these locations are adjacent to sites of known HPAI outbreaks among poultry in South Korea, making these sites ideal for surveillance^10^. From the 7942 fecal samples, 126 AIVs were isolated through the standard chicken egg isolation method^11,12^ (Table 1). Eighteen viruses were isolated in 2009, 4 in 2010, 18 in 2011, 1 in 2012, and 85 in 2013 (Table 1). The annual AIV isolation rate ranged from 0.42 to 5.14%, and the total virus isolation rate was 1.59% (Table 1). The location with the highest number of isolates was Cheonsuman Bay in Chungnam, South Korea (Fig. 1a).

**Table 1:** Isolation rates of AIVs from wild migratory bird fecal samples in South Korea, winter 2009‒2013.

Year	No. tested	No. of RT-PCR positive (%)	No. of virus isolates (%)
2009	350	18 (5.14)	18 (5.14)
2010	950	4 (0.42)	4 (0.42)
2011	1784	18 (1.01)	18 (1.01)
2012	1353	1 (0.07)	1 (0.07)
2013	3505	85 (2.43)	85 (2.43)
Total	7942	126 (1.59)	126 (1.59)

Percentages are relative to the total per year.

### HA and NA subtype combinations

Reverse transcription polymerase chain reaction (RT-PCR) using full-length influenza A virus universal primers was performed to determine the HA and NA subtypes of the isolates^13^. In the course of the 5-year study, we were able to identify the HA and NA subtypes of 118 out of 126 isolates (Table 2). In particular, we were able to isolate the H1‒H7 and H9‒H11 HA subtypes, and the N1‒N3, N5, and N7‒N9 NA subtypes of influenza virus from the fecal matter of wild migratory birds. The distributions of the HA and NA subtypes isolated per year are shown in Fig. 1b,c.

**Table 2:** Distribution of influenza A virus HA and NA subtypes among 126 avian influenza virus isolates.

Subtype	N1	N2	N3	N4	N5	N6	N7	N8	N9	NX	Total
H1	11	–	2	–	–	–	–	2	–	–	15
H2	–	2	4	–	–	–	–	–	2	1	9
H3	–	–	–	–	–	–	–	2	–	–	2
H4	–	1	–	–	–	–	–	–	–	1	2
H5	1	11	9	–	–	–	5	2	–	–	28
H6	17	25	1	–	2	–	–	3	–	–	48
H7	–	–	–	–	–	–	1	–	–	–	1
H8	–	–	–	–	–	–	–	–	–	–	0
H9	–	3	–	–	–	–	–	–	–	–	3
H10	5	–	–	–	–	–	1	–	–	1	7
H11	–	–	5	–	–	–	–	–	1	5	11
Total	34	42	21	0	2	0	7	9	3	8	**126**

**Figure 2:**
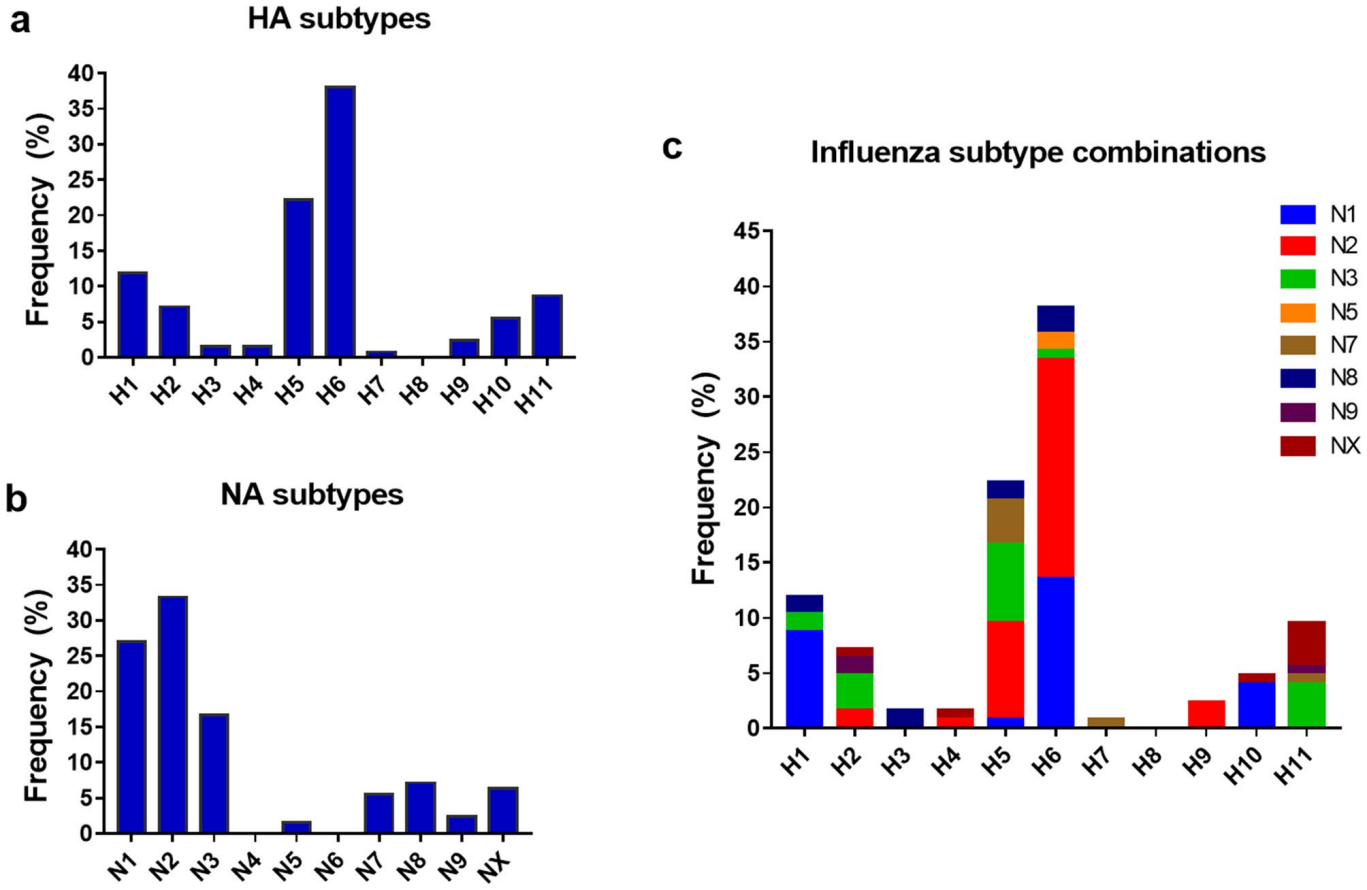
Overall frequency of hemagglutinin (HA) and neuraminidase (NA) subtypes of avian influenza viruses (AIVs) isolated from the fecal matter of wild migratory birds in Korea (2009–2013). The frequencies of (**a**) HA subtypes, (**b**) NA subtypes, and (**c**) HA and NA combinations of AIV isolates were calculated relative to the total number of isolates (n = 126). NX: unknown NA subtypes.

Over the 5-year sampling period, H6 (38.1%), H5 (22.2%), and H1 (11.9%) were the most frequently isolated HA subtypes, followed by H11 (8.7%), H2 (7.1%), H10 (5.6%), H9 (2.4%), H3 (1.6%), H4 (1.6%), and H7 (0.8%) (Table 2; Fig. 2a). Viruses belonging to the H8 and H12‒H16 subtypes were not detected. The most frequently detected NA subtypes were N2 (33.3%), N1 (27%), and N3 (16.7%), followed by N8 (7.1%), N7 (5.6%), N9 (2.4%), and N5 (1.6%) (Table 2; Fig. 2b). Viruses belonging to the N4 and N6 subtypes were not detected. However, the NA subtypes of 8 samples were not identified (NX: 6.3%) possibly due to mutations on the sites targeted by our NA primer sets. In total, 28 combinations of HA and NA subtypes were isolated (Table 2; Fig. 2c), and the most frequently isolated subtype was H6N2 which accounted for 19.8% (n = 25) of all isolates, followed by H6N1 (13.5%, n = 17), H1N1 and H5N2 (at 8.7% each with n = 11 per subtype), and H5N3 (7.1%, n = 9). Certain HA subtypes were found in combination with only one NA subtype: H3 combined with only N8; H4 with N2; H7 with N7; and H9 with N2. Based on the HA and NA sequencing data, no co-infection with two or more AIV subtypes was found in any sample. There was also high variability among the isolated AIV subtypes and the isolation rates per year.

### HA and NA sequence comparison of avian influenza isolates

To determine the genetic relationship of the isolates with HPAI HA, we compared the HA and NA genes of representative avian influenza isolates with known sequences. BLAST analysis of the HA and NA gene segments of avian influenza isolates revealed that most of them were similar to Eastern Asia genetic strains circulating in China, Korea, Japan, and Mongolia (Supplementary Table S1). All the H6- and H11-type isolates were found to have HA genes closely related to strains from the Republic of Georgia and the Netherlands (Supplementary Table S1). In our analysis, the HA gene nucleotide sequences of avian influenza isolates were similar to those of low-pathogenicity influenza strains and did not match any high-pathogenicity influenza strains.

### Phylogenetic analyses of H6- and N2-subtype isolates

**Figure 3:**
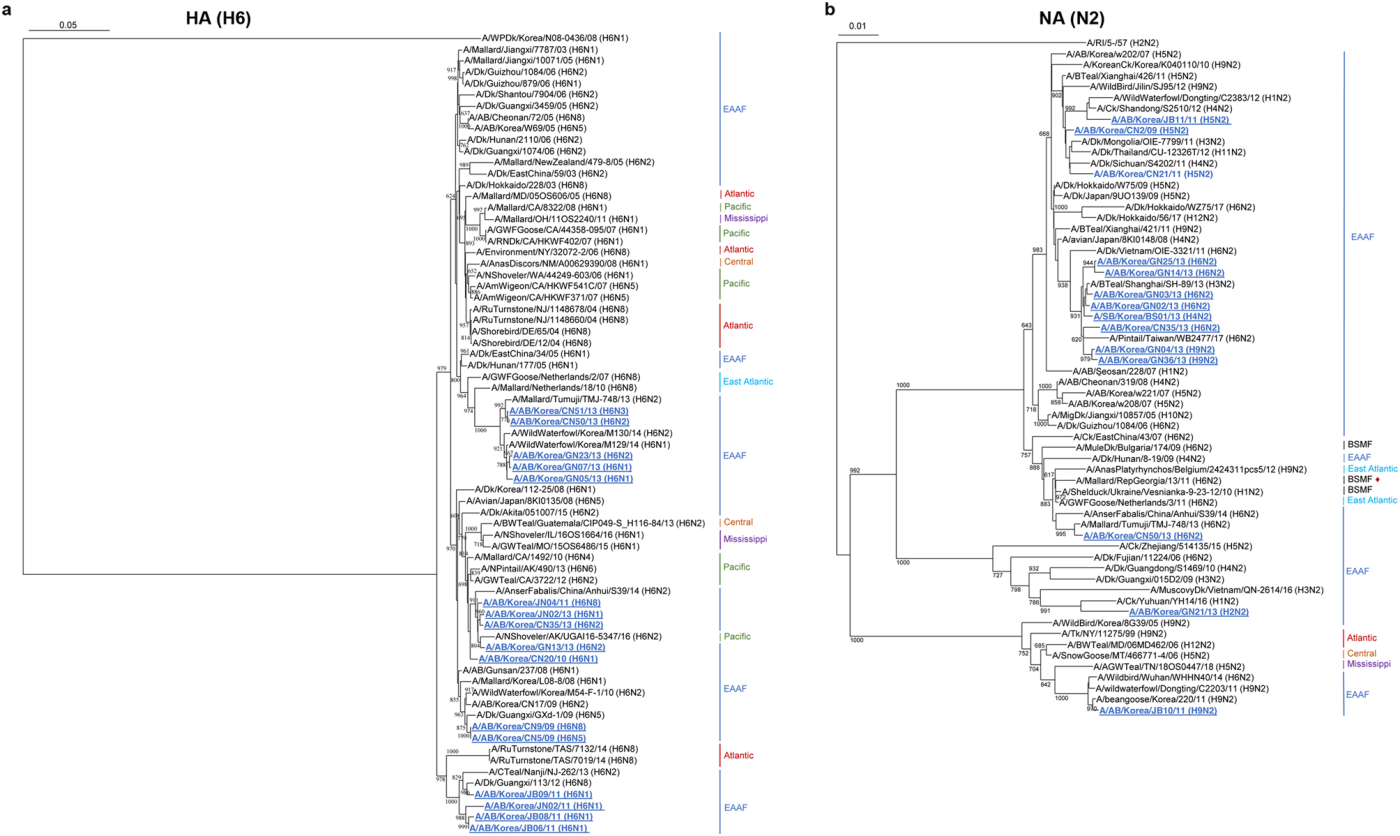
Phylogenetic analyses of representative H6- and N2-subtype avian influenza fecal isolates in South Korea in 2009–2013. The assembled (**a**) H6-subtype hemagglutinin and (**b**) N2-subtype neuraminidase coding sequences of representative isolates (blue, underlined) were compared to different isolates from various locations. Wildfowl migratory flyways that cover these locations are indicated. Bootstrap values ≥ 600 are shown. *BSMF* Black Sea-Mediterranean flyway, *EAAF* East Asian-Australasian flyway. Red diamond: the Republic of Georgia is at the intersection of the BSMF, Central Asian, and East Africa-West Asian flyways.

Given the frequency of H6-subtypes among our isolates and, we next compared representative H6 isolates from our study with previously reported H6 isolates from different locations via phylogenetic analysis. In general, our isolates clustered with those obtained from wild birds in different locations along the EAAF, particularly from China and Japan (Fig. 3a). Notably, some of our samples clustered with a 2016 isolate from the Pacific flyway, and some samples clustered with isolates from the East Atlantic flyway obtained from earlier years (2007 and 2010). These findings indicate that the genetic variants of H6-subtype AIVs that circulate in South Korea is generally confined to the EAAF. However, some of the H6 genetic variants in 2009–2013 may have been carried to different flyways. Genetic variants from other flyways have also contributed to the diversity of H6-subtype AIVs in South Korea in 2009–2013.

We also compared representative N2 isolates with previously reported isolates from various locations through phylogenetic analysis. In general, most of our isolates clustered with those of the Eurasian lineage (Fig. 3b). However, one isolate, A/AB/Korea/JB10/11(H9N2), along with other samples from China, clustered with isolates from the North American flyways obtained at an earlier year (2006). This indicates an event between 2006 and 2011 that carried the North American N2 genetic variant(s) to Eurasian flyways.

## Discussion

Since the zoonotic emergence that caused lethal outbreaks in poultry and humans in 1997, HPAI H5N1 virus strains have been established in domestic and wild bird populations^14–16^. HPAI outbreaks in domestic birds have taken place in all regions of the world between January 2013 and May 2021, and the most affected regions were Asia, Africa, and Europe^17^. Since 2003, South Korea has reported several HPAI outbreaks, mainly caused by H5N1, H5N8, and H5N6 viruses, and migratory birds are proposed to be the sources of these HPAI strains^18,19^. Previously, only the H5 subtypes were associated with serious infectious disease; however, strains of other HA subtypes (e.g., H6, H7, H9, and H10) have recently infected humans and have shown pandemic potential^20–22^.

In this surveillance study, we collected a total of 7942 fecal samples from stopover sites of wild migratory birds in South Korea. While we did not find the HPAI H5 subtypes (H5N1 and H5N8) that broke out in South Korea, we were able to isolate 126 samples of LPAI viruses, including those belonging to the H5, H6, H7, and H9 subtypes. There was high variability in isolation rates and subtype frequencies across the years. In particular, we had a very low isolation rate in 2012 despite collecting more samples that year than in 2009 and 2010. Isolation rates and AIV variability ultimately depend on the migration patterns of the birds that carry the AIVs. This may, in turn, be affected by shifts in seasonal patterns and other environmental factors^23^. The timing of sampling may also affect isolation rate, as this will be affected by the bird species present in the stopover sites, with certain species (e.g. Anseriformes and Charadriiformes) more likely to carry AIVs across flyways than others^24^. Compositions of the population (e.g., frequency of juveniles), which is also influenced by timing and environmental factors, may also affect AIV diversity^25,26^. However, for this study, we did not identify the species sources of the fecal samples, so we could not know for certain whether the bird population at the time of sampling affected our AIV isolation rates. These factors should be considered in future long-term surveillance studies, so that we may further understand the dynamics of AIVs among migratory waterfowl.

The most frequently isolated HA subtype was H6, which accounted for 38.1% (n = 48) of the isolates, with H6N2 and H6N1 being the most prevalent. Our results are in line with a study that reported that H6N2 and H6N1 were the most commonly isolated H6-subtype IAVs isolated from migratory and domestic birds in a surveillance study in South Korea in 2008 to 2009^27^. Likewise, a number of studies in other locations, including China, North America, and Europe, have reported that H6-subtype AIVs are the most commonly detected AIV subtypes among wild and domestic fowl^28–31^. Given that H6-subtype AIVs are classified as LPAI, the implications of the prevalence of this subtype to animal and human health are still unclear. However, AIVs of the H6 subtype may reassort with AIVs of other subtypes and act as precursors to HPAIs^8^, thereby emphasizing the need for continuous surveillance and characterization of LPAIs in the wild.

As expected, most of our H6- and N2-subtype isolates clustered with samples from the EAAF, especially with isolates obtained in China and Japan. In a previous study, five H6 subtypes (H6N1, H6N2, H6N5, H6N6, and H6N8) were found to have circulated in Eastern and Southern China from 2002 to 2010 and 2009 to 2011, with H6N2 and H6N6 being predominant^28,32^. Four H6 subtypes (H6N1, H6N2, H6N5, and H6N8) circulated in South Korea between 2009 and 2013. South Korea is one of the migratory stopover sites on the EAAF, and our results imply that migratory birds passing through Korea along the EAAF do not carry all the AIV subtypes circulating in China. A few of our H6 samples also clustered with samples from the Pacific and the East Atlantic flyways obtained from different years, and one of the representative N2 samples clustered with isolates from the North American flyways. These findings indicate that intermingling among migratory birds occasionally occurs in breeding and wintering sites where flyways intersect and facilitates the movement of genetic variants from the EAAF to other flyways and vice versa.

Here, we report the distribution of circulating AIVs in migratory water birds in South Korea during 2009‒2013. The most prevalent AIVs were of the H6 subtype, and the circulating AIVs in this period were similar to low-pathogenicity strains along the EAAF. Because wild birds are able to transmit pathogenic influenza viruses to local fowl, surveillance studies such as this are important so that we may be able to predict and mitigate the effects of AIVs on animal and human health.

## Methods

### Sample collection and virus isolation

Through surveillance studies from winter of 2009 to 2013, a total of 7942 fecal samples were collected from stopover sites of wild migratory birds in South Korea (Table 1, Fig. 1a). Fecal samples were stored in transport medium composed of phosphate buffered saline (PBS) and glycerol (50%) with antibiotics (1000 U/ml of penicillin G and polymyxin B, 500 U/ml of nystatin, 250 µg/ml of gentamicin, 60 µg/ml of ofloxacin, and 200 µg/ml of sulfamethoxazole). The collected samples were stored at − 80 °C until analysis.

Samples were suspended in antibiotic-supplemented PBS and inoculated into the allantoic cavity of 10-day-old embryonated chicken eggs^12^. The allantoic fluid was collected. Turbid fluid was assumed to be contaminated with bacteria or fungi and was discarded. All viral isolates were collected from the first egg passage. Viral presence was identified through hemagglutination assay using 0.5% chicken red blood cells^11^.

### RT-PCR and sequence analysis

Viral gene amplification was performed as previously described^25^. Briefly, viral RNA was extracted from the allantoic fluid of embryonated chicken eggs infected with the supernatant of fecal samples using the RNeasy Mini Kit (Qiagen, Valencia, CA). For HA and NA gene subtyping, one-step RT-PCR was carried out using the One Step RT-PCR Kit (Qiagen, Valencia, CA) with HA- or NA-specific universal primer sets designed and described in a previous study^13^. The RT-PCR reactions were prepared according to manufacturer instructions. Each 50-μL reaction contained 1.5 μL of each primer (20 pmol/μL) and 2 μL of viral RNA (1 pg–2 µg). Reverse transcription was performed at 50 °C for 30 min, and standard PCR was performed with an initial denaturation at 94 °C for 10 min, followed by 35 cycles of 94 °C for 30 s, 56 °C for 30 s, and 72 °C for 2 min, and a final extension at 72 °C for 10 min. After purification with the QIAquick Gel Extraction Kit (Qiagen, Valencia, CA), the amplified gene segments were commercially sequenced at Cosmogenetech Co. (Seoul, South Korea) using the high-throughput DNA Analyzer (Applied Biosystems 3730xl DNA Analyzer, Thermo-Fisher Scientific, Waltham, MA), which is based on the Sanger sequencing technology. Full-length sequences (HA: 1710 bp; NA: 1410 bp) were assembled using the Lasergene sequence analysis software package (DNASTAR, Madison, WI). A BLASTn query was performed to identify the HA and NA subtypes of the isolates based on the obtained sequences.

### Phylogenetic analyses of H6 and N2 coding sequences

The assembled complete coding sequences of representative H6- and N2-subtype isolates were aligned using CLUSTAL V^33^. Rooted phylogenetic trees were prepared using the neighbor-joining method (1000 bootstrap replicates) and then visualized with the NJ Plot software^34^. Accession numbers of HA and NA sequences of isolates included in the phylogenetic analyses are listed in Supplementary Table S2.

## Supplementary Information

Supplementary Information.

## Data Availability

All data generated or analysed during this study are included in this published article.
