# Supplementary material for: Surveillance of avian influenza viruses from 2009 to 2013 in South Korea
Source: Sci Rep. 2021 Dec 14;11:23991. doi: 10.1038/s41598-021-03353-1 (PMC8671502; doi:10.1038/s41598-021-03353-1)
Supplement: Supplementary file 1 — Supplementary Information. [file 41598_2021_3353_MOESM1_ESM.pdf]

SUPPLEMENTARY INFORMATION

Surveillance of avian influenza viruses from 2009 to 2013 in South Korea

Jeong-Hyun Nam[1,2,†], Erica Españo[1,†], Eun-Jung Song[1,3], Sang-Mu Shim[1,2], Woonsung Na[3], Seo-Hee Jeong[1], Jiyeon Kim[1], Jaebong Jang[1], Daesub Song[1,*], Jeong-Ki Kim[1,*]

[1] *Department of Pharmacy, Korea University College of Pharmacy, Sejong 30019, Republic of Korea*

[2] *Division of Acute Viral Diseases, Center for Emerging Virus Research, National Institute of Health, Korea Disease Control and Prevention Agency, Cheongju, Chungbuk 28159, Republic of Korea*

[3] *Laboratory Animal Medicine, College of Veterinary Medicine, Chonnam National University, Gwangju 61186, Republic of Korea*

[†] These authors contributed equally to this work.

* Corresponding authors: Jeong-Ki Kim; e-mail: jkfrancis@korea.ac.kr; tel.: +82 44 860 1613; fax: +82 44 860 1609, and Daesub Song; e-mail: sds1@korea.ac.kr; tel.: +82 44 860 1625; fax: +82 44 860 1609

Supplementary Table S1. GenBank sequence identification of hemagglutinin and neuraminidase genes from representative avian influenza isolates

Isolate	Subtype	Virus with the highest sequence identity*			
		Hemagglutinin	Identity (%)	Neuraminidase	Identity (%)
#54	H5N2	A/baikal teal/Xianghahai/426/2011 (H5N2)	99%	A/duck/Jiang Xi/1286/2005 (H5N2)	100%
#99	H6N5	A/spot-billed duck/Korea/625/2008 (H6N1)	97%	A/duck/Guangxi/GXd-1/2009 (H6N5)	99%
#114	H5N3	A/mallard/Bavaria/1/2005 (H5N2)	97%	A/mallard/Italy/43/01 (H7N3)	89%
#146	H6N8	A/spot-bill duck/korea/546/2008 (H6N1)	97%	A/duck/Hokkaido/228/2003 (H6N8)	97%
#153	H11NX	A/spotbill duck/Xuyi/6/2005 (H11N2)	97%	A/duck/Hokkaido/327/2009 (H1N3)	96%
#289	H1N3	A/duck/Hebei/843/2005 (H1N2)	97%	A/duck/Hokkaido/327/2009 (H1N3)	97%
#312	H5N2	A/duck/Jiang Xi/1850/2005 (H5N2)	97%	A/garganey/Altai/1213/2007(H5N2)	97%
#337	H6N2	A/spot-billed duck/Korea/528/2008 (H6N8)	98%	A/duck/Tsukuba/9/2005 (H2N2)	97%
#430	H3N8	A/aquatic bird/Hong Kong/399/99 (H3N8)	98%	A/duck/Eastern China/90/2004 (H3N8)	98%
#817	H11N9	A/mallard/Netherlands/17/2007 (H11N8)	98%	A/Baikal teal/Hongze/14/2005 (H11N9)	93%
#842	H3N8	A/avian/Japan/8KI0067/2008 (H3N8)	99%	A/avian/Japan/8KI0067/2008 (H3N8)	100%
#1074	H6N1	A/avian/Japan/8KI0195/2008 (H6N8)	99%	A/mallard/California/6768/2009 (H1N1)	99%
#1723	H5N2	A/duck/Thailand/CU-12205C/2012(H5N8)	98%	A/duck/Korea/A14/2008 (H5N2)	98%
#1764	H5N3	A/duck/Hokkaido/W280/2014(H5N3)	97%	A/duck/Hokkaido/327/2009 (H1N3)	98%
#2322	H5N3	A/duck/Hokkaido/W280/2014(H5N3)	98%	A/aquatic bird/Korea/w16/2005 (H5N3)	95%
#2435	H5N3	A/duck/Hokkaido/WZ20/2014(H5N2)	97%	A/wild duck/Korea/PJ25/2006 (H1N3)	96%
#2631	H9N2	A/wild waterfowl/Dongting/C4316/2012(H9N2)	99%	A/snow goose/Montana/466771-4/2006 (H5N2)	98%
#2647	H5N2	A/duck/Hokkaido/W240/2014(H5N3)	98%	A/duck/Korea/A14/2008 (H5N2)	98%
#3025	H1N3	A/goose/Italy/296426/2003 (H1N1)	95%	A/duck/Hokkaido/327/2009 (H1N3)	97%
#3074	H6N8	A/aquatic bird/Korea/CN20/2009 (H6N1)	98%	A/avian/Japan/8KI0062/2008 (H3N8)	98%
KU893	H1N1	A/duck/Hainan/Q47/2012 (H1N2)	97%	A/duck/Mongolia/675/2010 (H1N1)	93%
KU959	H4N2	A/spot-billed duck/Shanghai/SH148/2014 (H4N6)	96%	A/baikal teal/Shanghai/SH-89/2013 (H3N2)	96%
KU984	H10N1	A/duck/Mongolia/709/2015 (H10N7)	96%	A/Aquatic bird/South Korea/SW1/2018 (H10N1)	98%
KU990	H10N7	A/migratory duck/Jiangxi/33158/2013 (H10N7)	95%	A/duck/Okayama/331111/2014 (H10N7)	98%

KU1020	H6N2	A/mallard/republic of Georgia/13/2011 (H6N2)	96%	A/wild waterfowl/Korea/M130/2014 (H6N2)	96%
KU1149	H9N2	A/duck/Hokkaido/K04/2014 (H9N2)	96%	A/baikal teal/Shanghai/SH-89/2013 (H3N2)	99%
KU1189	H6N1	A/wild bird/Wuhan/CDHN09/2015 (H6N2)	97%	A/wild bird/Wuhan/WHHN16/2014 (H1N1)	96%
KU1469	H1N1	A/duck/Hainan/Q47/2012 (H1N2)	98%	A/mallard/Republic of Georgia/4/2012 (H1N1)	99%
KU1482	H11N3	A/duck/Jiangxi/22620/2012 (H11N9)	99%	A/duck/Saga/411114/2013 (H10N3)	96%
KU1487	H6N1	A/Anser fabalis/China/Anhui/L221/2014(H6N1)	98%	A/wild bird/Wuhan/WHHN16/2014 (H1N1)	96%
KU1594	H7N7	A/wild bird/Jiangxi/34458/2013(H7N7)	98%	A/duck/Okayama/331111/2014 (H10N7)	98%
KU1599	H6N2	A/aquatic bird/Korea/CN20/2009(H6N1)	98%	A/tundra swan/shimane/3211A001/2011(H5N2)	97%
KU1679	H5N3	A/duck/Hokkaido/WZ20/2014(H5N2)	96%	A/duck/Jiangxi/33629/2013 (H10N3)	89%
KU1735	H5N7	A/duck/Hokkaido/W280/2014(H5N3)	97%	A/duck/Okayama/331111/2014 (H10N7)	98%
KU1758	H1N8	A/duck/Hainan/Q47/2012(H1N2)	97%	A/duck/Mongolia/30/2011 (H3N8)	96%
KU1875	H6N3	A/Anser fabalis/China/Anhui/L221/2014(H6N1)	97%	A/duck/Viet Nam/QT-1877/2014 (H5N3)	98%
KU2261	H1N1	A/duck/Hainan/Q47/2012(H1N2)	97%	A/mallard/Republic of Georgia/4/2012 (H1N1)	99%
KU2556	H6N8	A/aquatic bird/Korea/CN20/2009 (H6N1)	97%	A/common shelduck/Mongolia/2185/2011 (H3N8)	98%

*The presented AIV isolates are the first hits upon BLASTn search of assembled hemagglutinin or neuraminidase coding sequences.

Supplementary Table S2. Genbank accession numbers of representative hemagglutinin (HA) and neuraminidase (NA) sequences.

Isolate	Subtype	HA	NA
**A/aquatic bird/Korea/CN5/2009**	H6N5	CY088572	CY088574
**A/aquatic bird/Korea/CN9/2009**	H6N8	CY098238	CY098240
**A/aquatic bird/Korea/CN20/2010**	H6N1	CY098230	CY098232
**A/aquatic bird/South Korea/GN36/2013**	H9N2	OK342149	OK342150
**A/shorebird/South Korea/BS01/2013**	H4N2	OK342151	OK342152
**A/aquatic bird/South Korea/GN02/2013**	H6N2	OK342153	OK342154
**A/aquatic bird/South Korea/GN03/2013**	H6N2	OK342155	OK342156
**A/aquatic bird/South Korea/GN04/2013**	H9N2	OK342157	OK342158
**A/aquatic bird/South Korea/GN05/2013**	H6N1	OK342159	OK342160
**A/aquatic bird/South Korea/GN07/2013**	H6N1	OK342161	OK342162
**A/aquatic bird/South Korea/GN14/2013**	H6N2	OK342163	OK342164
**A/aquatic bird/South Korea/GN21/2013**	H2N2	OK342165	OK342166
**A/aquatic bird/South Korea/GN25/2013**	H6N2	OK342167	OK342168
**A/aquatic bird/South Korea/CN35/2013**	H6N2	OK342169	OK342170
**A/aquatic bird/South Korea/CN21/2011**	H5N2	OK342171	OK342172
**A/aquatic bird/South Korea/CN50/2013**	H6N2	OK342173	OK342174
**A/aquatic bird/South Korea/CN51/2013**	H6N3	OK342175	OK342176
**A/aquatic bird/South Korea/JB06/2011**	H6N1	OK342177	OK342178
**A/aquatic bird/South Korea/JB08/2011**	H6N1	OK342179	OK342180
**A/aquatic bird/South Korea/JB09/2011**	H6N1	OK342181	OK342182
**A/aquatic bird/South Korea/JN02/2013**	H6N8	OK342183	OK342184
**A/aquatic bird/South Korea/JB10/2011**	H9N2	OK342185	OK342186
**A/aquatic bird/South Korea/JB11/2011**	H5N2	-	OK342187
**A/aquatic bird/South Korea/JN02/2011**	H6N1	OK342188	OK342189
**A/aquatic bird/South Korea/JN04/2011**	H6N8	OK342190	OK342191
**A/aquatic bird/South Korea/GN13/2013**	H6N2	OK342192	-
